# Prognostic value of performance status in metastatic renal cell carcinoma patients receiving tyrosine kinase inhibitors: a systematic review and meta-analysis

**DOI:** 10.1186/s12885-019-5375-0

**Published:** 2019-02-22

**Authors:** Yawei Xu, Yuanyuan Zhang, Xianhao Wang, Jiaqi Kang, Xiaoqiang Liu

**Affiliations:** 1 Department of Urology, Tianjin Medical University General Hospital, 154 Anshan Road, Heping District, Tianjin, 300052 China; 2 Department of Thyroid Surgery, The First Affiliated Hospital of Zhengzhou University, Zhengzhou, Henan China

**Keywords:** Metastatic renal cell carcinoma, Performance status, Prognosis, Mete-analysis

## Abstract

**Background:**

The association between performance status (PS) and the prognosis of metastatic renal cell carcinoma (mRCC) patients receiving tyrosine kinase inhibitors (TKIs) remains controversial. The aim of this study is to evaluate the prognostic value of PS in mRCC patients treated with TKIs.

**Methods:**

Electronic databases were searched to identify the studies that had assessed the association between pretreatment PS and prognosis in mRCC patients receiving TKIs. Hazard ratios (HRs) and 95% confidence interval (CI) for overall survival (OS) and progression-free survival (PFS) from eligible studies were used to calculate combined HRs. The heterogeneity across the included studies was assessed by Cochrane’s *Q* test and *I*^*2*^ statistic. The Begg’s funnel plot and Egger’s linear regression teats were used to evaluate the potential publication bias. The meta-analysis was performed with RevMan 5.3 and Stata SE12.0 according to the PRISMA guidelines.

**Results:**

A total of 6780 patients from 19 studies were included in this meta-analysis. The results showed that a poor PS was an effective prognostic factor of both OS (pooled HR: 2.08, 95% CI: 1.78–2.45) and PFS (pooled HR: 1.51, 95% CI: 1.20–1.91). Subgroup analysis revealed that poor PS significantly associated with poor OS and PFS in studies using Karnofsky PS scale (OS, pooled HR: 2.20, 95% CI: 1.65–2.94; PFS, pooled HR: 1.74, 95% CI: 1.19–2.56), conducted in Asia (OS, pooled HR: 2.25, 95% CI: 1.71–2.95; PFS, pooled HR: 1.73, 95% CI: 1.14–2.64) and Newcastle-Ottawa Scale score of 8 (OS, pooled HR: 2.61, 95% CI: 1.92–3.55; PFS, pooled HR: 2.43, 95% CI: 1.36–4.33).

**Conclusions:**

This study suggests that a poor PS is significantly associated with poor prognosis in mRCC patients receiving TKIs.

## Background

Renal cell carcinoma (RCC) is the most common cancer of kidney in adults [1]. Nearly half of RCC patients eventually progress to metastatic RCC (mRCC), and the 5-year survival rate of these patients is poor [2, 3]. The molecular mechanisms of the pathogenesis have been widely investigated and has promoted the development of targeted agents in the past few decades [4]. Tyrosine kinase inhibitors (TKIs), which target the vascular endothelial growth factor pathway, have been approved for the first-line or later line of treatment for mRCC [5–7]. The TKIs, such as sorafenib, sunitinib, axitinib and pazopanib, have been consistently demonstrated in clinical trials to prolong both overall survival (OS) and progression-free survival (PFS) in patients with mRCC [6–8]. However, these targeted agents have provoked marked changes in the management of RCC, and new predictive and prognosis clinical markers are required.

Many previous studies have demonstrated that the prognosis of mRCC patients treated with TKIs varies greatly after treatment [7, 9], so it is important to assess which patients may benefit from TKIs before treatment. Performance status (PS) is used to quantify quickly the general well-being of people with illness and their ability to perform activities of daily living [10]. It is usually a proxy measure estimated by clinician and influences the decision to apply treatment regiments [11–13]. PS has been identified as an independent prognostic factor in several cancers, such as bladder cancer [12], lung cancer [11] and liver cancer [13]. Previous reports have shown that a poor PS predicts poor survival of patients with metastatic RCC [14–16]. However, the prognostic value of PS in patients with mRCC treated with TKIs is controversial. Bamias et al. found that PS was an independent prognostic factor (HR: 3.04, 95% CI: 1.46–6.33, *p* = 0.003) for mRCC patients receiving TKIs [17], and several other studies have drawn similar conclusions [18–20]. But there were also studies have shown no significant relationship between PS and prognosis [21–26], such as a retrospective study of 257 patients by Hwang et al. (HR: 1.71, 95% CI: 0.74–3.95, *p* = 0.21) [26].

Furthermore, the association between PS and survival outcomes in patients with mRCC receiving TKIs has not been previously reviewed. Therefore, we conducted a systematic review and meta-analysis to evaluate the prognostic value of pretreatment PS in mRCC patients receiving TKIs.

## Methods

### Systematic search strategy

We conducted a systematic search of electronic databases, including PubMed, Embase, Web of Science and the Cochrane Library (updated 1 May 2018), to identify all relevant studies. The studies were searched using the terms “renal cell carcinoma or kidney cancer” AND “performance status or PS” AND “prognosis, survival or outcomes”. The language of publication was limited to English. In cases of multiple reports from the same series, we used the most recent one. And we also searched the lists of eligible articles. Two investigators independently completed all the work the search strategy, filtered the titles and abstracts of all articles according to the following eligibility criteria.

### Eligibility criteria

The eligibility criteria of studies included in this meta-analysis were listed as: (1) retrospective studies focused on the value of PS in predicting prognosis in patients with mRCC; (2) patients who received TKIs therapy mRCC; (3) hazard ratios (HRs) and 95% confidence intervals (CIs) for overall survival (OS) or progression-free survival (PFS) should be reported in the articles or have enough information to calculate them; (4) pretreatment PS measured before administering TKIs.

### Data extraction

The data was extracted by two investigators and the other two were responsible for checking. All authors have discussed the disagreements until a consensus was reached. A standardized form was created and used to extract available data from all eligible publications including the first author’s name, publication year, region, study period, the number of patients, duration of follow-up, age, gender (male/female), tumor histology, type of PS, HRs, 95% CIs and its *P* value. If multiple HRs were presented in the original articles, we extracted the estimates from the largest adjusted model to reduce the risk of possible unmeasured confusion.

### Quality assessment

Two investigators independently assessed the quality of all included studies. The Newcastle-Ottawa Scale (NOS) system was designed to evaluated the quality of non-randomized studies in meta-analysis [27]. It assessed study quality by 3 classifications including selection, comparability and outcome with a total of 9 stars. Studies with a total score of ≤5 stars, 6–7 stars, and 8–9 stars were considered to be of low quality, intermediate quality, and high quality respectively. All included studies had an intermediate or high quality according to NOS.

### The assessment of PS

Eastern Cooperative Oncology Group performance status (ECOG PS) scale and Karnofsky performance status (KPS) scale are the two most widely used measurement instruments to evaluate the performance status of mRCC patients [28–31]. The KPS scale rating ranges from 100, indicating that all functions can perform normal daily activities without clinical evidence (symptoms or signs), to 0, which means death [10]. ECOG introduced the ECOG PS scale with only 6 points, which was a more simplified scale ranging from 0 (fully active) to 5 (death) [32]. In view of the fact that the cut-off values classified in practical applications were not completely consistent, we defined poor PS as the group with lower KPS scale scores or the group with higher ECOG PS scale scores, while others were considered to be good PS.

### Statistical analysis

We performed a formal meta-analysis of OS and PFS. HRs with 95% CIs from each study were used to calculate combined HRs. Cochrane’s *Q* test and Higgins *I*^*2*^ statistic were used to assess the heterogeneity across the studies. The studies with *P* > 0.1 and *I*^*2*^ < 50% were considered indicative of significant heterogeneity. If no significant heterogeneity was found, a pooled estimate was calculated with a fixed effect model; or, a random effect model was used. The Begg’s funnel plot and Egger’s linear regression teats were used to evaluate the potential publication bias. A sensitivity analysis was performed to evaluate the stability of the results and to reduce the effect of individual studies on final conclusions. Two-tailed value of *P* <  0.05 was considered statistically significant. The meta-analysis was performed with RevMan 5.3 and Stata SE12.0 (Stata Corp LP, College Station, TX, USA) according to the PRISMA guidelines [33].

## Results

**Fig. 1:**
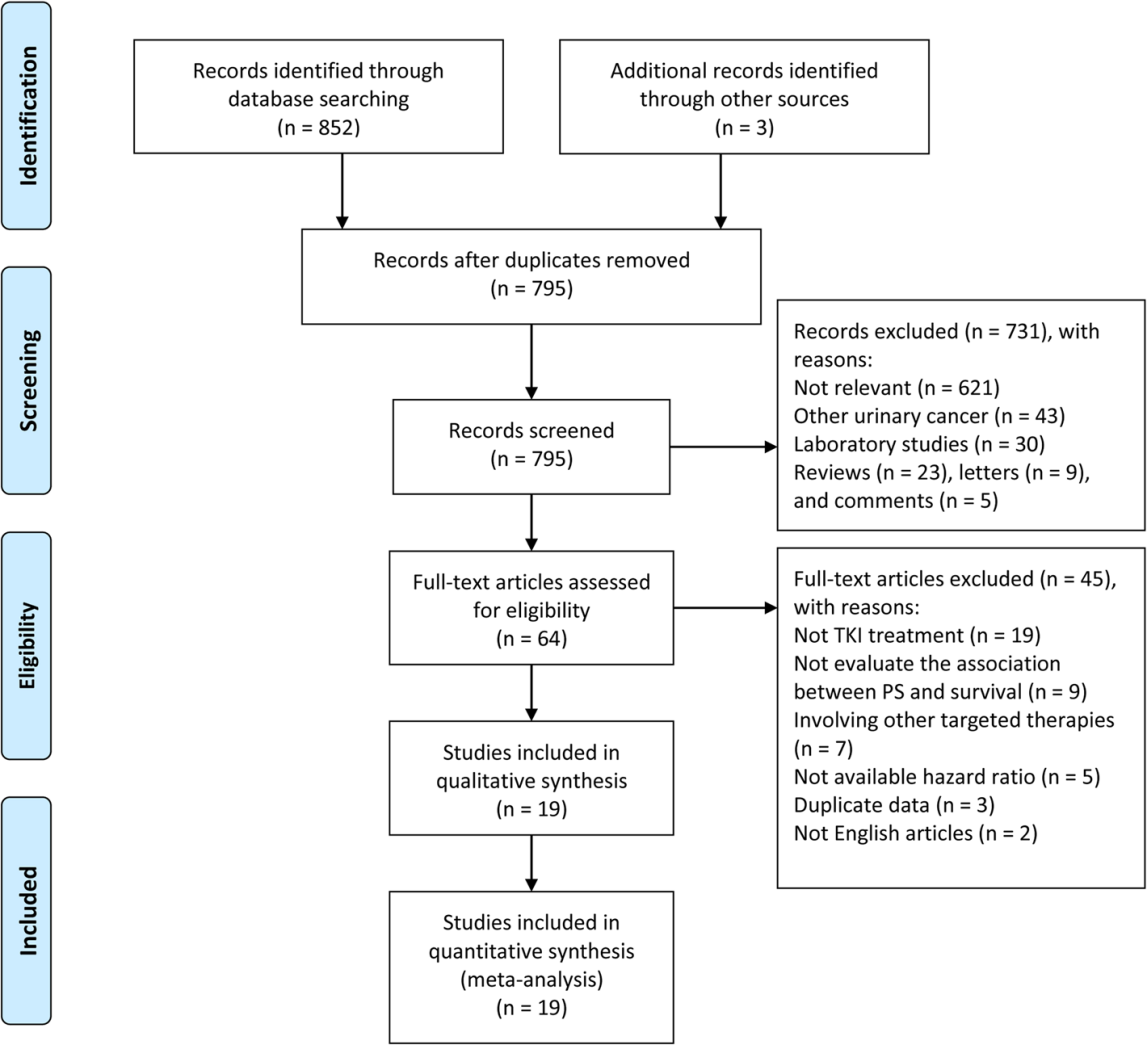
Preferred Reporting Items for Systematic Reviews and Meta-analysis flow diagram: search and study selection process for this review

A total of 852 articles were identified from electronic databases (PubMed, Embase and the Cochrane Library) and 3 additional studies were identified from reference lists. Sixty duplicate articles were removed. After a careful review of titles and abstracts, 731 articles were excluded for not relevant, other urinary cancer, laboratory studies, reviews, letters and comments. After assessing the full text of the remaining 64 articles, 45 articles were excluded for some specific reasons, including not TKI treatment, not evaluate the association between PS and survival outcome, involving other targeted therapies, not available hazard ratio, duplicate data and not English articles. Finally, 19 retrospective cohort studies [17–26, 34–42] were included in the following meta-analysis. Figure 1 shows the full screening procedure.

### The features of included studies

The characteristics of the included studies are shown in Table 1. These 19 articles were published between 2010 and 2018. Among them, 11 were published before 2015 and 8 were published in the past 3 years. Seven, nine and three studies were conducted in Asian, Europe and America, respectively. Sample size of included studies ranged from 39 to 4543 patients, and a total of 6780 patients were included. All trials were conducted in adult patients who received TKIs. The mean (median) age of the subjects ranged from 57 to 68.8 years, and the percentage of included males ranged from 65.6 to 82.9%. Clear cell carcinoma accounted for 62.5 to 100% of all pathological types. Eleven of the 19 studies used ECOG PS scale to assess PS and another eight used KPS scale. Six articles reported the prognostic value of PS for both OS and PFS in patients with mRCC receiving TKIs, 11 articles only reported OS and 2 other articles only reported PFS.

**Table 1:** Characteristics of included studies

Study	Region	Study Period	No. of patients	Follow-up (months)	Age (years)	Gender (male/female)	Tumor histology			Type of PS scale	Survival	NOS
							ccRCC	non-ccRCC	Unknown			
Bamias 2010	UK	2006–2009	109	15.8 (0.1–31.5)	59 (30–79)	80/29	100	7	2	ECOG PS	OS	7
You 2011	Korea	2006–2009	78	10	59 (34–79)	57/21	78	–	–	KPS	OS, PFS	8
Steffens 2011	Germany	2005–2010	116	21 (9.5–30.3)	61 ± 11.1	79/37	98	18	–	ECOG PS	OS, PFS	7
Abel 2011	USA	2004–2009	75	15 (10.5–23.0)	60.0 (23–80)	54/21	55	11	9	ECOG PS	OS	8
Seidel 2012	Germany	2005–2011	119	NA	61 (27–72)	84/35	106	13	–	ECOG PS	OS	6
Powles 2014	UK	2006–2009	98	NA	59 (37–78)	75/23	98	–	–	ECOG PS	OS	8
Poprach 2014	Czech	2007–2012	319	15	62 (45–77)	221/98	309	NA	NA	ECOG PS	OS, PFS	7
Park 2014	Korea	2006–2011	83	18 (1–62)	57 (33–80)	60/23	83	–	–	KPS	OS, PFS	7
Kust 2014	Croatia	2008–2013	41	40 (5–48)	60	34/7	41	–	–	ECOG PS	OS, PFS	6
Gore 2015	USA	2005–2007	4543	13.6 (1–71.3)	59 (19–89)	3364/1179	4010	532	1	ECOG PS	OS	8
Shin 2015	Korea	2006–2011	182	34.6 (2.3–171.7)	56.9 (33–85)	132/50	182	–	–	KPS	OS, PFS	6
Wang 2016	China	2006–2015	282	37	58 (19–83)	200/82	233	49	–	KPS	OS	8
Rausch 2016	Germany	2003–2014	88	34.6 (26.4–42.2)	68.8 (30.8–81.5)	69/19	81	6	6	ECOG PS	OS	6
Chrom 2016	Poland	2010–2014	266	46.1 (41.2–51.0)	61 (22–85)	180/86	248	18	–	ECOG PS	OS	6
Artac 2016	Turkey	1998–2012	104	20.8 (1.8–55.5)	57 (29–88)	77/27	65	15	24	KPS	PFS	8
Furukawa 2016	Japan	2009–2011	39	15.1	61 (36–77)	27/12	39	–	–	KPS	PFS	7
Hwang 2017	Korea	2007–2013	56	20.2 (1.5–78.3)	60 (37–88)	44/12	51	5	–	KPS	OS	7
Teishima 2017	Japan	2007–2016	118	NA	64 (40–84)	97/21	102	16	–	ECOG PS	OS	7
Zucca 2018	Brazil	2008–2014	64	18.5	57 (30–81)	42/22	51	13	–	KPS	OS	7

*ccRCC* clear cell renal cell carcinoma, *PS* performance status, *NOS* Newcastle-Ottawa score, *KPS* Karnofsky performance status, *ECOG PS* Eastern Cooperative Oncology Group performance status, *NA* not available

The cut-off value of the 6 studies using KPS scale to evaluate the patient’s PS was 80%, and the other 2 studies were 70% [37, 42]. Of the 11 studies using ECOG PS scale, 8 had a cut-off value of 1 and the other 3 had a cut-off value of 2 [17–19].

### Survival outcomes

**Fig. 2:**
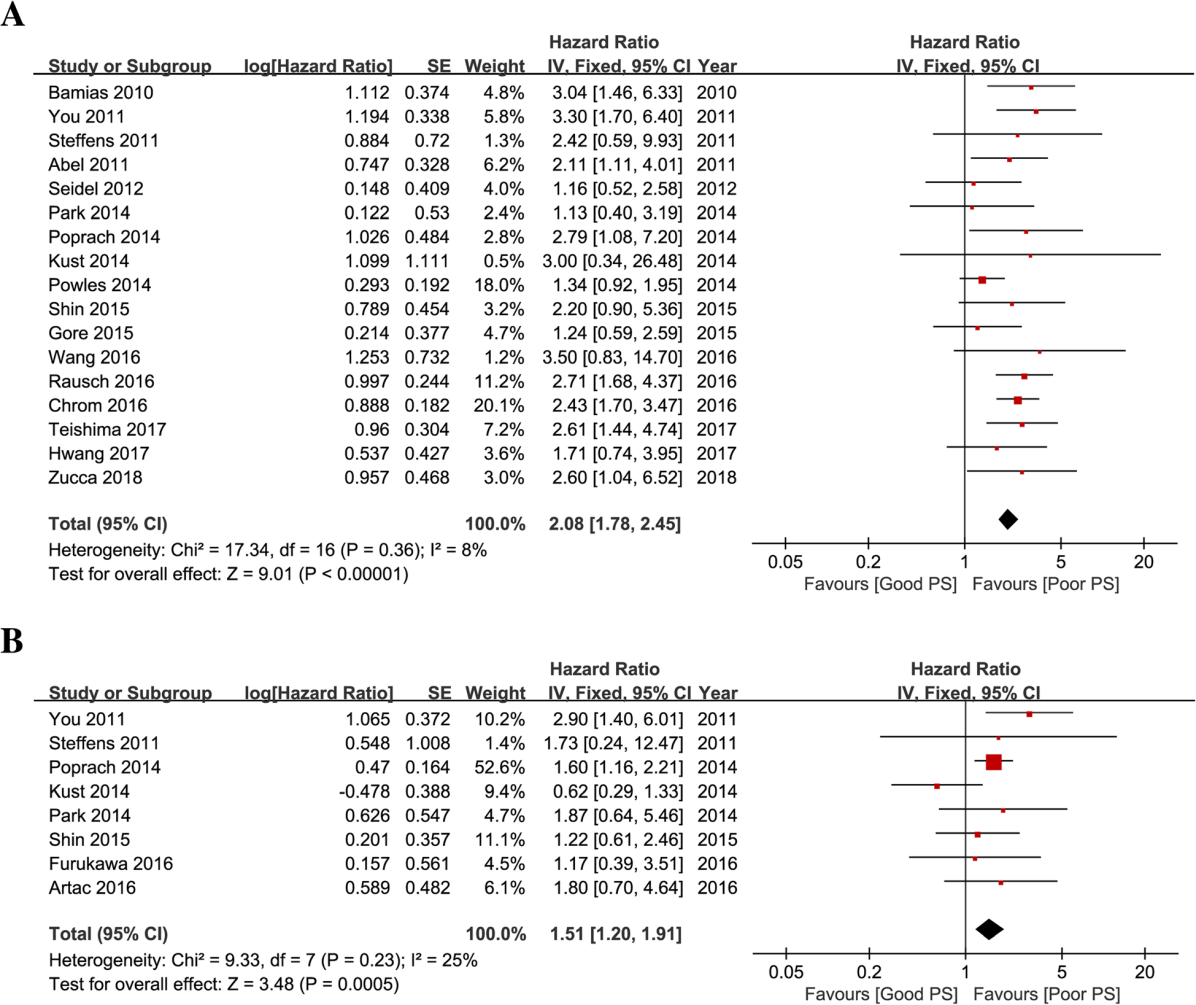
Forest plot showing the effects of PS on **a** overall survival **b** progression-free survival in mRCC patients receiving TKIs

Prognostic outcomes, including OS and PFS, were quantitatively synthesized. The impact of PS on OS was investigated in 17 studies including 6637 mRCC patients receiving TKIs. The forest plot (Fig. 2a) shows that poor PS was significantly associated with poor OS (pooled HR: 2.08, 95% CI: 1.78–2.45). The Cochrane *Q* test (*Chi*^*2*^ = 17.34, *P* = 0.36) and *I*^*2*^ test (*I*^*2*^ = 8%) did not show significant heterogeneity.

The impact of PS on PFS was investigated in 8 studies including 962 mRCC patients receiving TKIs. The forest plot (Fig. 2b) shows that poor PS was significantly associated with poor PFS (pooled HR: 1.51, 95% CI: 1.20–1.91). The Cochrane *Q* test (*Chi*^*2*^ = 9.33, *P* = 0.23) and *I*^*2*^ test (*I*^*2*^ = 25%) did not show significant heterogeneity.

### Subgroup analysis

Table 2 summarizes the results of subgroup analysis according to the type of PS scale, study setting, year of publication and NOS score. Pooled analysis of studies with KPS associated with worse OS (pooled HR: 2.20, 95% CI: 1.65–2.94; *P* = 0.26, *I*^*2*^ = 24%) and PFS (pooled HR: 1.74, 95% CI: 1.19–2.56; *P* = 0.50, *I*^*2*^ = 0) than ECOG PS. Subgroup analysis according to study setting revealed that Asian studies associated with worse OS (pooled HR: 2.25, 95% CI: 1.71–2.95; *P* = 0.24, *I*^*2*^ = 26) and PFS (pooled HR: 1.73, 95% CI: 1.14–2.64; *P* = 0.34, *I*^*2*^ = 11) than European and American studies. Pooled HRs for survival outcome stratified by the NOS score showed that worse OS (pooled HR: 2.61, 95% CI: 1.92–3.55; *P* = 0.90, *I*^*2*^ = 0) and PFS (pooled HR: 2.43, 95% CI: 1.36–4.33; *P* = 0.43, *I*^*2*^ = 0) in studies with an NOS score of 8. Due to the small number of literatures, no further subgroup analysis can be conducted on the studies that focus on PFS.

**Table 2:** Subgroup analyses for the association between PS and the survival

Subgroup analysis	OS					PFS				
	No. of studies	Pooled HR (95% CI)	*P* value	Heterogeneity		No. of studies	Pooled HR (95% CI)	*P* value	Heterogeneity	
				*I*^*2*^ (%)	*P*				*I*^*2*^ (%)	*P*
Overall	17	2.08 (1.78–2.45)	< 0.00001	8	0.36	8	1.51 (1.20–1.91)	0.0005	25	0.23
Type of PS scale										
KPS	6	2.20 (1.65–2.94)	< 0.00001	24	0.26	5	1.74 (1.19–2.56)	0.005	0	0.50
ECOG PS	11	2.03 (1.68–2.46)	< 0.00001	6	0.39	3	1.39 (1.04–1.87)	0.03	61	0.08
Study setting										
Asia	6	2.25 (1.71–2.95)	< 0.00001	26	0.24	4	1.73 (1.14–2.64)	0.01	11	0.34
Europe	8	1.95 (1.56–2.43)	< 0.00001	28	0.20	4	1.42 (1.08–1.88)	0.01	44	0.15
America	3	2.25 (1.43–3.53)	0.0005	0	0.93	0	–	–	–	–
Year of publication										
Before 2015	11	1.79 (1.44–2.22)	< 0.00001	17	0.28	6	1.51 (1.18–1.94)	0.001	44	0.11
After 2015	6	2.49 (1.97–3.15)	< 0.00001	0	0.95	2	1.50 (0.73–3.07)	0.27	0	0.56
NOS score										
6	5	2.07 (1.57–2.74)	< 0.00001	28	0.24	2	0.90 (0.54–1.50)	0.67	40	0.20
7	7	1.80 (1.40–2.32)	< 0.00001	19	0.29	4	1.57 (1.18–2.10)	0.002	0	0.92
8	5	2.61 (1.92–3.55)	< 0.00001	0	0.90	2	2.43 (1.36–4.33)	0.003	0	0.43

*OS* overall survival, *PFS* progression-free survival, *PS* performance status, *KPS* Karnofsky performance status, *ECOG PS* Eastern Cooperative Oncology Group performance status, *NOS* Newcastle-Ottawa score, *CI* confidence interval

### Publication bias

**Fig. 3:**
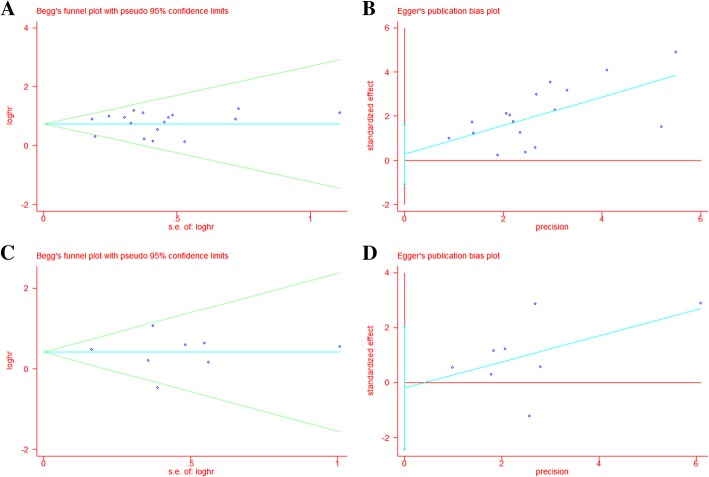
Funnel plots based on overall survival **a** Begg’s test **b** Egger’s test; and progression-free survival **c** Begg’s test **d** Egger’s test

Begg’s funnel plots test and Egger’s test were used to assess the publication bias in this meta-analysis (Fig. 3). Both Begg’s funnel plots test (OS: *P* = 0.773, PFS = 0.711) and Egger’s test (OS: *P* = 0.671, PFS = 0.834) verified that no obvious publication bias exists.

### Sensitivity analysis

**Fig. 4:**
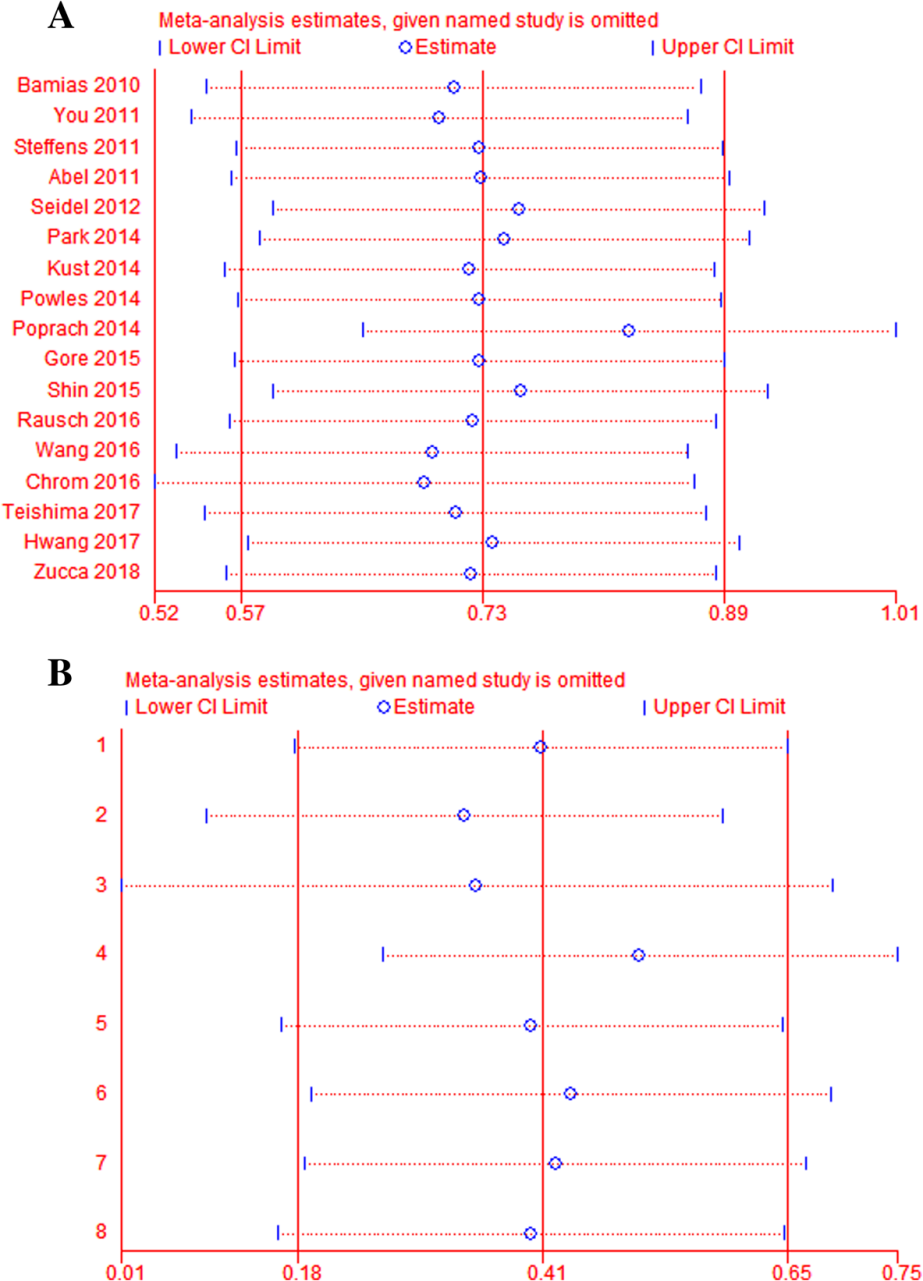
Sensitivity analysis in this mate-analysis. **a** sensitivity analysis for overall survival; **b** sensitivity analysis for progression-free survival

A sensitivity analysis was performed to evaluate the stability of the results and to reduce the effect of individual studies on final conclusions (Fig. 4). The test suggested that the pooled results did not tend to alter when a study was excluded.

## Discussion

Since applying TKIs to the management of mRCC patients, the prognosis of these patients has been significantly improved compared to that in the era of cytokine therapy [43, 44]. The introduction of these drugs has induced a dramatic paradigm shift in the treatment of mRCC. These molecular targeted agents need novel factors that precisely reflect susceptibility to TKIs, thereby providing individualized risk-directed treatment for patients with mRCC. To date, several model systems predicting the prognosis of patients with mRCC in the era of immunotherapy have been reported [14, 45, 46]; in particular, the Memorial Sloan-Kettering Cancer Center (MSKCC) scoring system is still applied to patients receiving TKIs era [17]. However, these models were based on traditional markers of risk, rather than molecular characteristics.

This meta-analysis based on currently available clinical evidence adjusted clinical and demographic variables that may have affected survival outcomes. We found that poor PS may significantly predict unfavorable prognostic outcomes in mRCC patients receiving TKIs and may play an important role in the management of mRCC patients. Although the International Metastatic Renal Cell Carcinoma Database Consortium (IMDC) [19] and MSKCC [17] risk models are being used to predict the prognosis of mRCC patients, PS is expected to be a simpler indictor for screening of mRCC patients who received TKIs in clinical practice.

PS was used to quickly quantify the general health status of the patient population and their ability to perform daily activities [11, 47]. It is usually a proxy measure estimated by the clinician or researcher and affects the decision to apply the management plan, especially in terms of conservative and non-conservative care and planning for self-care. PS has been reported to be associated with the survival outcomes of several malignancies, such as bladder cancer [12], hepatocellular carcinoma [13], and non-small-cell lung cancer [11]. PS has been regarded as a key determinant of the malignant tumor patients’ ability to undergo therapy.

Many previous studies have consistently identified that PS was a significant prognostic factor in patients with RCC [14–16]. In a retrospective study of 670 patients with RCC [14], median survival time was 2.7 , 6.1 , 10.6 and 14.4 months for patients with Karnofsky PS (KPS) of 60, 70, 80 and 90%, respectively (*P* <  0.0001). As well, another study identified ECOG PS as an independent prognostic factor for survival in a multivariate analysis of 782 patients of mRCC [48]. However, the prognostic value of PS in patients with mRCC treated with TKIs was controversial.

There are some limitations in our meta-analysis. First, all the included studies in this meta-analysis were retrospective, which may lead to selection bias. High-quality prospective researches needed to further investigation in this field. Second, the simple size of partial eligible was relatively small. The large-scale studies are necessary to achieve more credible results in the future. Finally, the potential heterogeneity might still exist. Although heterogeneity was not significantly from the results of meta-analysis and subgroup analysis, but the cut-off values across the included studies were not completely consistent, which might lead to unknown heterogeneity. Therefore, more uniform standards should be established to increase homogeneity between the studies.

## Conclusions

To conclude, the present meta-analysis demonstrates that poor PS may significantly predict unfavorable prognostic outcomes in mRCC patients receiving TKIs. Therefore, PS may play an important role in the management of mRCC patients. However, in order to better evaluate the prognostic value of PS in mRCC patients treated with TKIs, additional prospective, large-scale, and homogeneous clinical studies will be needed in the future.

## Abbreviations

CI: Confidence interval
ECOG PS: Eastern Cooperative Oncology Group performance status
HRs: Hazard ratios
KPS: Karnofsky performance status
mRCC: metastatic renal cell carcinoma
MSKCC: Memorial Sloan-Kettering Cancer Center
NOS: Newcastle-Ottawa Scale
OS: Overall survival
PFS: Progression-free survival
PS: Performance status
RCC: Renal cell carcinoma
TKIs: Tyrosine kinase inhibitors
